# Factors affecting intradiscal pressure measurement during in vitro biomechanical tests

**DOI:** 10.1186/1748-7161-10-S1-O19

**Published:** 2015-01-19

**Authors:** Jaelle Tremblay, Jean-Marc Mac-Thiong, Vladimir Brailovski, Yvan Petit

**Affiliations:** 1 Centre de Recherche de l'Hopital du Sacre-Coeur, Canada; 2 Ecole de Technologie Superieure, Canada

## Objective

To assess the reliability of intradiscal pressure measurement during in vitro biomechanical testing. The variability of measurements will be assessed for repeated measures by considering the effect of specimens and of freezing/thawing cycles.

## Materials and methods

Thirty-six functional units from 8 porcine spines (S1: T7-T8, S2: T9-T10, S3: T12-T11, S4: T14-T13, S5: L1-L2 and S6: L3-L4) have been used. Before the experiments, intervertebral discs were measured in the frontal and sagittal planes to locate the center of the discs. A catheter was then inserted upto the center of the disc. Finally, a fiber optic pressure sensor was inserted into the catheter. The specimens were divided into 3 groups: fresh (F), after one freeze/thaw cycle (C1), and after 2 freeze/thaw cycles (C2). These groups were divided in two, depending on whether the specimens were axially loaded at 400 N or not. Ten consecutive measurements were performed for each case. Statistical analyses were achieved to evaluate the variability of measurements for repeated measures, porcine specimens and vertebral levels using MANOVA. The difference between freeze/thaw cycles were analysed with U Mann-Whitney test (P ≤ 0.05).

## Results

With no axial loading, the intradiscal pressure was found to be 365 mbar for F group, 473 mbar for C1 group, and 391 mbar for C2 group. When 400 N axial load was applied, intradiscal pressure was 10610 mbar for F group, 10132 mbar for C1 group, and 12074 mbar for C2 group. Statistical analyses showed a significant influence of the porcine specimen (p<0.001), with and without axial loading, and of the vertebral level with (p=0.048) and without loading (p<0.001). The intradiscal pressure was also significantly different between the freeze/thaw cycles, with (p<0.001) and without (p=0.033) axial loading. Repeated measurements (p = 0.93 without loading and p = 0.83, with loading) did not show significant variation.

## Conclusion

The results show that freezing/thawing cycles and inter-specimen variability can affect intradiscal pressure measurements. These findings suggest using the specimen as its own control when evaluating 2 configurations (i.e. with and without spinal instrumentation) during in vitro biomechanical tests.

## Significance

Physicians need to take into consideration the impact of spinal instrumentation strategy (type and level of instrumentation) on the load distribution. Intradiscal pressure measurement during testing of instrumented spine segments could help evaluate the resulting intervertebral loading. Consequently, it is important to validate such measurements, considering different constraints representative of in vitro biomechanical testing conditions.

